# Preventing Antibiotic Resistance in the Wild: A New End Point for Environmental Risk Assessment

**DOI:** 10.1289/ehp.120-a321a

**Published:** 2012-08-01

**Authors:** Julia R. Barrett

**Affiliations:** Julia R. Barrett, MS, ELS, a Madison, WI–based science writer and editor, has written for *EHP* since 1996. She is a member of the National Association of Science Writers and the Board of Editors in the Life Sciences.

Human and veterinary antibiotics enter the environment through wastewater effluent, agricultural use of manure and treated sewage for fertilizer, and leakage from waste storage facilities. This antibiotic pollution may exert selective pressure on bacteria to develop drug resistance, which is especially concerning if it develops in pathogenic bacteria. A new study weighs known bacterial antibiotic sensitivities against the backdrop of antibiotic concentrations measured in the environment and suggests that resistance in clinically relevant bacteria may not be kept in check by current risk assessment action levels [*EHP* 120(8):1100–1106; Tello et al.].

Ciprofloxacin, erythromycin, and tetracycline were selected for the analysis from among the approximately 150 compounds for which minimum inhibitory concentrations (MICs) have been reported for different bacteria. An MIC is the amount of antibiotic needed to inhibit bacterial growth. Each of the three antibiotics affects a wide range of bacteria and represents a distinct class of antibiotics used in humans and animals. Concentrations of each antibiotic in various environmental compartments, or media, had been documented previously.

Current guidelines for environmental risk assessment reflect the phase I action limits of the International Cooperation on Harmonisation of Technical Requirements for Registration of Veterinary Medicinal Products. This program, which evaluates the safety of veterinary drugs, calls for environmental risk assessment of antibiotics whose concentrations exceed 1 ppb in aquatic compartments or 100 ppb in terrestrial compartments. These guidelines have been implemented in regulations in the United States, Europe, Japan, and Australia.

**Figure:**
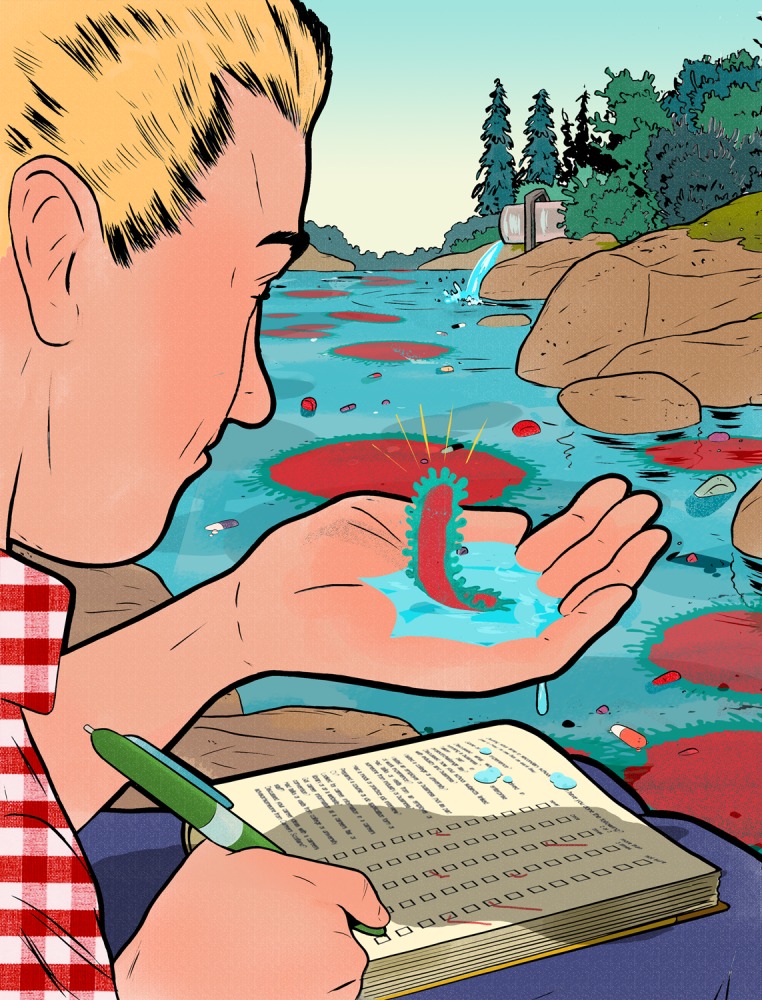
www.jasonschneider.com

The researchers compared information on antibiotic levels in the environment with information on the MICs of clinically relevant bacteria to determine if current levels of antibiotic pollution might be high enough to promote the development of resistance. Within aquatic compartments, only a small fraction of the 27 bacterial genera included in the study were predicted to be affected by environmental concentrations of antibiotics, and results suggested that the phase I action level would protect against resistance. However, current levels of antibiotic pollution in terrestrial compartments—particularly in river sediments, liquid manure, and farmed soil—may be high enough to favor the evolution of bacterial resistance.

The conclusions may be limited by long-term selective pressure and by the fact that MIC tests do not represent environmental conditions. Furthermore, the authors did not address bioavailability or the potential influences of antibiotic mixtures, metals, or disinfectants. Nevertheless, the potential for antibiotic pollution to increase antibiotic resistance in clinically relevant bacteria has important implications for public health and environmental health policy, and the authors demonstrate a possible framework for answering important outstanding questions about the environmental impact of antibiotics.

